# Behavioral activation versus treatment as usual in naturalistic sample of psychiatric patients with depressive symptoms: a benchmark controlled trial

**DOI:** 10.1186/s12888-018-1820-x

**Published:** 2018-07-27

**Authors:** Kaisa E. Luoto, Lars H. Lindholm, Vesa Paavonen, Antti Koivukangas, Antero Lassila, Esa Leinonen, Olli Kampman

**Affiliations:** 1 Faculty of Medicine and Life Sciences, University of Tampere, PB 100, FI-33014 Tampere, Finland; 2 Department of Psychiatry, Tampere University Hospital, PB 2000, FI-33521 Tampere, Finland; 3 Department of Psychiatry, Seinäjoki Hospital District, Huhtalantie 53, 60220 Seinäjoki, Finland

**Keywords:** Behavioral activation, Depressive disorder, Functional outcome, Naturalistic sample

## Abstract

**Background:**

More systematic use of evidence-based brief therapies is needed in the treatment of depression within psychiatric care. The aim of this study was to explore the impact of behavioral activation therapy (BA) for patients with depressive symptoms in a routine clinical setting of secondary psychiatric care.

**Methods:**

The BA-treated intervention group (*n* = 242) comprised patients with depressive symptoms (Beck Depression Inventory (BDI) score ≥ 17 at baseline). The control group (*n* = 205) patients received treatment as usual in the same catchment area. The groups were matched at baseline using BDI and Alcohol Use Disorders Identification Test scores and inpatient/outpatient status. The groups were compared at 6-, 12- and 24-month follow-up points on functional outcome (Global Assessment of Functioning scale), service use, dropout and deaths. The Montgomery–Åsberg Depression Rating Scale was used to assess depressive symptoms in the intervention group.

**Results:**

The estimated difference in GAF score between intervention and control group patients was significant at 12- and 24-months follow-up points in favor of intervention group (GAF score difference 4.85 points, *p* = 0.006 and 5.71 points, *p* = 0.005, respectively; estimate for patient group 2.26, *p* = 0.036). The rates of dropout, mortality and service use were similar between the groups. Among the intervention group patients, the estimated improvement in MADRS score compared to baseline was statistically significant throughout the follow-up (*p* < 0.001 at all follow-up points).

**Conclusions:**

The systematic use of BA among secondary psychiatric care depressive patients provides encouraging results despite the patients had various comorbid non-psychotic disorders.

**Trial registration:**

ClinicalTrials.gov, Identifier NCT02520271, Release Date: 06/27/2015, retrospectively registered.

## Background

Depression is a leading cause of burden and often reduces the level of functioning [1]. Recurrent episodes and comorbidity with other psychiatric symptoms make treatment challenging. Comorbidities of depression are very common in secondary psychiatric care [2] According to the guidelines of American Psychiatric Association and National Institute for Health and Care Excellence [3, 4], non-pharmacological therapeutic intervention should be included in the treatment of depression. Often, chosen therapeutic methods are not standardized or evidence based, although there are well-documented therapeutic options available [5, 6]. Evidence-based brief protocols should be used more widely; these are easy to implement among the usual psychiatric services [7].

It is not possible or necessary to offer every patient long-term therapies. Previous research indicates that some brief therapies are effective [8, 9]. One example of an evidence-based brief intervention for treatment of depression is behavioral activation (BA) [10, 11]. BA is an application of cognitive-behavioral therapy and includes a maximum of 24 appointments. The aim is to explore the patient’s actions in relation to his or her environment. The avoidance of certain actions, and the simultaneous avoidance of negative or otherwise uncomfortable feelings, can result in negative consequences and exacerbate depressive symptoms. BA focuses on helping the patient change this negative circle by changing his or her behavior. To our knowledge, there are no studies exploring the effectiveness of BA in a psychiatric naturalistic patient population. Patients with common comorbidities, such as problematic alcohol use and suicidal behavior, are often excluded from study samples.

The Ostrobothnia Depression Study (ODS) was a benchmark controlled trial (BCT) [12] which in practice means an observational intervention study. BCT can be used to assess the impact of clinical intervention in routine settings (in contrast to RCTs which usually assess the specific intervention in ideal settings). ODS aimed at developing a systematic model for assessment and treatment of patients with depression and comorbid non-psychotic disorders [13]. The purpose was to increase the effectiveness of psychiatric services by implementing a systematic use of brief therapy and improving the process of recognizing patients who could benefit from this type of therapy. The aim of the present study is to explore the benefits of BA in a heterogeneous group of depressed patients in a naturalistic setting and to compare BA with treatment as usual (TAU) in terms of functional recovery, service use, dropout and mortality.

## Methods

### Study setting and participants

According to the idea of BCTs, the aim of ODS was to study the impact of the selected intervention in real life setting of psychiatric secondary services. The comparisons were made with a control group representing as similar patient population as possible treated with TAU methods in the psychiatric services of the same area. This setting allowed the assessment of differences in outcomes between health care units treating similar patients with different methods (more structured brief intervention vs. treatment as usual).

ODS patients were recruited in five psychiatric outpatient clinics and one psychiatric hospital ward in the South Ostrobothnia hospital district of Finland (population 200,000) during October 2009–October 2013. Consecutive patients who were referred to adult psychiatric services because of depressive symptoms, anxiety, self-destructiveness, insomnia or substance-related problems were screened using the Beck Depression Inventory (BDI, version 1A) [14] . Those with a BDI score ≥ 17 (corresponding to at least moderate-level depression) at the screening phase were included in the intervention group (*n* = 242). In addition to having depressive symptoms, these patients had various other psychiatric diagnoses which is typical in psychiatric secondary services. Patients with a likely or verified psychotic disorder (other than psychotic depression, International Classification of Diseases [ICD]-10, F2*.**) [15] or organic brain disease were excluded.

The control group (*n* = 205) was recruited from the South Ostrobothnia hospital district database of psychiatric outpatient clinics not participating in the ODS study (time period: October 2009–December 2012) and from the same psychiatric hospital ward before the start of the ODS (i.e., before 30 September 2009). Patients with a new referral to adult psychiatric services were selected in chronological order if their BDI score was ≥17 at admission and the Alcohol Use Disorders Identification Test (AUDIT) [16] score at admission was available. Patients who had participated in the ODS study or had a likely or verified psychotic disorder (other than psychotic depression, ICD-10, F2*.**) or organic brain disease were excluded. The control group patients were matched with the intervention group patients by clustering according to the current psychiatric hospital contact (inpatient/outpatient), AUDIT score in four categories (0–7, 8–10, 11–19 and 20–40 points) and BDI score in two categories (17–29 or 30–60 points). Roughly 1650 patients´ files were screened before the amount of patients in the control group was considered to be close enough to the intervention group. The main characteristics of intervention and control group patients at the baseline are presented in Table 1. The characteristics were mainly similar between the groups, only the baseline GAF score (*p* = 0.035) and the frequencies of personality disorders as a secondary psychiatric diagnosis (*p* = 0.037) were statistically significantly different between the groups. The psychiatric diagnoses were register based and determined by the responsible doctor in previous treatment contacts.

**Table 1:** The main characteristics of patients at baseline

	Intervention group	Control group	
	n (%)	n (%)	p^e^
Number of patients	242	205	
Female / Male	148 (61.2) / 94 (38.8)	111 (54.1) / 94 (45.9)	0.135
Outpatient / Inpatient	189 (78.1) / 53 (21.9)	156 (76.1) / 49 (23.9)	0.615
Register based^f^ clinical diagnosis (ICD-10):			
Primary psychiatric diagnosis			0.103
Depressive disorder (F32.x)	98 (40.5)	89 (50.0)	
Recurrent depressive disorder (F33.x)	92 (38.0)	48 (27.0)	
Bipolar disorder (F31.x)	19 (7.9)	17 (9.6)	
Anxiety disorders (F40–43.x)	9 (3.7)	20 (9.6)	
Other disorders	18 (7.4)	–	
Secondary psychiatric diagnosis			
Panic disorder (F41.x)	26 (10.7)	16 (7.8)	0.296
Phobic and other anxiety disorders (F40,42,43.x)	22 (9.1)	22 (10.8)	0.550
Personality disorder (F6x.x)	9 (3.7)	17 (8.4)	0.037
Tertiary psychiatric diagnosis			
Anxiety disorder (F4x.x)	12 (5.0)	5 (2.5)	0.168
Personality disorder (F6x.x)	8 (3.3)	10 (4.9)	0.393
Alcohol dependence (current)	47 (19.4)	n/a	
Use of other substance (last 12 months)	22 (9.1)	n/a	
	mean (SD)	mean (SD)	p
Age (years)	38.8 (12.2)	39.5 (12.7)	0.567
Baseline MADRS^a^	23.2 (6.7)	n/a	
Baseline BDI^b^	27.9 (7.3)	28.8 (7.9)	0.214
Baseline AUDIT^c^	10.7 (9.9)	9.8 (9.0)	0.319
Baseline GAF^d^	45.9 (10.7)	43.6 (11.7)	0.035

^a^Montgomery–Åsberg Depression Rating Scale

^b^Beck Depression Inventory

^c^Alcohol Use Disorders Identification Test

^d^Global Assessment of Functioning scale

^e^between groups (chi-square test)

^f^Register based diagnoses determined by the responsible doctor in previous treatment contacts

Antidepressive medication was used for 239 (98.8%) patients in the intervention group and for 203 (99.5%) patients in the control group (cumulative doses: 28.0 mg, standard deviation [SD] 20.6 mg vs. 26.7 mg, SD 21.2 mg fluoxetine equivalents [17, 18] , respectively). Sixty-six (27.3%) patients in the intervention group and 72 (35.1%) patients in the control group were using antipsychotic medication (cumulative doses: median 62.5 mg, interquartile range [IQR] 93.75 vs. 125 mg, IQR 187.5 chlorpromazine equivalents [19], respectively).

Information on somatic comorbidities was collected during the baseline assessment of the intervention group patients and from the psychiatric patient files of the control group patients. Thirteen patients in the intervention group and one patient in the control group had hypertension (I10). Four patients in the intervention group had diabetes mellitus type 1 (ICD-10, E10). Eight patients in the intervention group and one patient in the control group had diabetes mellitus type 2 (E11). In the intervention group, five patients had metabolic syndrome (E66), three patients had coronary heart disease (I25) and two patients had sequelae of cerebrovascular disease (I69). The patients in either group didn’t have any current infections.

### Baseline assessment

For the ODS intervention group, the baseline assessment was based on the Cube Method which is an integrated assessment method for comorbid psychiatric and substance use disorders in clinical settings; the method was developed by Kampman and Lassila in the South Ostrobothnia hospital district [20, 21]. Systematic assessment helps to target suitable treatment for each patient. The Cube Method was used to decide which patients would be additionally treated with motivational interviews. The baseline psychiatric diagnostic evaluation was performed using the Mini International Neuropsychiatric Interview (MINI) [22]. Depressive symptoms were rated using the Montgomery–Åsberg Depression Rating Scale (MADRS) [23] and level of functioning was assessed using the Global Assessment of Functioning scale (GAF) [24]. All evaluations were conducted by experienced psychiatrists or trained research nurses. The use of psychotropic medications was recorded using a paper-and-pencil diary. The study protocol is described in more detail in the Clinical Trials registry [13].

For the control group, all data were collected retrospectively from the hospital district patient registers. The diagnoses had been determined by the responsible doctor and were not confirmed using structured diagnostic interviews. GAF scores were estimated according to case notes by a trained research nurse supervised by a research doctor. In cases of insufficient information, scoring was omitted.

The GAF estimates were calibrated by prior rating of five test patients and comparing the possible differences between the raters (one research doctor and two research nurses). After the calibration process, the inter-rater correlations were strong (*r* > 0.9).

### Procedures and follow-up

In the intervention group, BA and antidepressive medication were used for 239 patients. Additionally, motivational interviews (MIs) [25] were used during the first appointments with patients having alcohol use problems (baseline AUDIT ≥11) to be able to better ensure the patients engagement to the treatment. BA and MIs were implemented by the regional staff (registered psychiatric nurses, psychiatric practical nurses and psychologists) responsible for the patient. The regional staff was systematically trained to use the selected interventions [7]. The minimum duration of BA was set at four appointments. On request, we received information about the completed BA sessions from the therapists of 54 patients. The median number of sessions was 6.5 (IQR 8.25). The decision to start the medication and the type of medication used was based on clinical evaluation (at baseline and at 6 weeks) by the responsible doctor. If the baseline MADRS score was 20 or more the medication was started and the dose was elevated if necessary, or the type of antidepressive medication was changed (from selective serotonin reuptake inhibitors to serotonin–norepinephrine reuptake inhibitors). The follow-up included appointments with a clinical research nurse at 6, 12 and 24 months and depressive symptoms (MADRS) and the level of functioning (GAF) were checked (among other things,  please see ClinicalTrials.gov Identifier NCT02520271).

All patients in the control group were treated in public psychiatric secondary care in the same organization and catchment area as the intervention group over the same time period. Control patients received TAU according to the protocols of the respective treatment unit and were followed-up according to the case notes at 6, 12 and 24 months by estimating GAF scores and obtaining information about possible alcohol use. In the area, psychiatric secondary care is always supervised by a specialized psychiatrist. The psychiatric TAU usually consists of regular individual visits to the outpatient clinic with varying frequency depending on each patient’s situation. Most often, a nurse, psychologist or social worker is responsible for the individual visits; patients meet a doctor only at the beginning and end of the treatment and whenever the treatment plan is evaluated. The TAU methods varied according to the educational background and therapeutic training of the responsible staff.

For both groups, information about the frequency of outpatient visits, number of hospital days and dropouts was collected from patient registers at 6, 12 and 24 months follow-up points and information on mortality was collected from baseline (October 1, 2009) until March 30, 2016. The register data covered all cases during the whole follow up in both intervention and control groups (*n* = 242 and *n* = 205, respectively).

### Statistical methods

The power calculations resulted in the following detectable limits for primary outcomes MADRS and GAF. Within intervention group including the current sample size, the paired analyses were able to detect a 1.7 point mean response difference in MADRS with a power of 0.8 and type I error probability of 0.05. Between intervention and control groups including the current number of pairs, the independent analyses were able to detect a 3.6 point mean response difference in GAF with a power of 0.8 and type I error probability of 0.05.

The independent samples t-test was used to compare the age distributions, GAF and AUDIT scores between the intervention and the control group at baseline. Chi-square tests were used to compare the frequencies of gender, inpatients and outpatients, psychiatric diagnoses and the patients hospitalized during the follow-up between the groups. A linear mixed model for repeated measures was used to analyze the changes in MADRS score from baseline to the three follow-up points in the intervention group patients and the changes in GAF score between the intervention and control groups. In the latter model, parameters time, patient group and an interaction term time*patient group were used in analyses. The Mann–Whitney U test was used to analyze antipsychotic medication doses and the number of days spent in hospital. The level of statistical significance was set at < 0.05. All calculations were performed using the software SPSS for Apple Macintosh version 24 (IBM SPSS Statistics, Armonk, NY) and PS: Power and Sample Size Calculation (version 3.1.2, Vanderbilt University, Nashville TN) [26].

## Results

### Depressive symptoms in the intervention group

Improvement of depressive symptoms in intervention group patients was analyzed using MADRS scores. The mean MADRS score for the intervention patients at baseline was 23.2 points (*n* = 228, SD 6.69), at 6 months 13.1 points (*n* = 156, SD 8.69), at 12 months 9.93 points (*n* = 135, SD 7.83) and at 24 months 8.31 points (*n* = 95, SD 7.58). The estimated change in MADRS score compared to baseline was statistically significant in every follow-up period. The results are presented in the Table 2.

**Table 2:** The results of linear mixed model for repeated measures of MADRS^a^ score in the intervention group

Follow-up period	N	Estimate^b^	95% CI	SE	*p*
0–6 months	156	−10.06	−11.58 - -8.54	0.77	< 0.001
0–12 months	135	−13.15	−14.64 - -11.65	0.76	< 0.001
0–24 months	95	−14.82	−16.35 - -13.29	0.77	< 0.001

^a^Montgomery-Åsberg Depression Rating Scale

^b^Estimated change in MADRS scores from baseline

### Level of functioning

At 12 months and 24 months follow-up points the estimated improvement in GAF score was significantly better in the intervention group patients (estimate for patient group 2.26, *p* = 0.036). At six months a similar difference was not found. For more detailed results please see Table 3. A sensitivity analysis was performed by excluding the patients with personality disorder as secondary clinical diagnoses (*n* = 44). The results were similar with the total sample analysis, with GAF estimates between intervention and control groups 0–6 months 2.84 (*p* = 0.057), 0–12 months 5.13 (*p* = 0.006), and 0–24 months 6.58 (*p* = 0.002).

**Table 3:** The results of linear mixed model for repeated measures of GAF^a^ score between the intervention and control groups

Follow-up period	Intervention (n)	Control (n)	Estimate^b^	95% CI	SE	*p*
0–6 months	167	159	2.65	−0.14 – 5.44	1.42	0.063
0–12 months	128	134	4.85	1.43–8.28	1.74	0.006
0–24 months	94	98	5.71	1.76–9.67	2.01	0.005

^a^Global Assessment of Functioning Scale

^b^Estimated difference in GAF score between intervention and control groups at the respective follow-up points

### Use of services

There were no between-group differences in number of outpatient visits (comparisons: no visits, one visit/month, two visits/month, more than two visits/month) during any of the follow-up periods (intervention group: *n* = 242, control group: *n* = 205; 0–6 months, *p* = 0.74; 6–12 months, *p* = 0.56; 12–24 months, *p* = 0.52). The need for hospitalization was measured in every follow-up period as “yes, patient was hospitalized” or “no, patient was not hospitalized.” The number of hospitalized patients was similar in the intervention and control groups (intervention group: n = 242, control group: n = 205) during all periods: 0–6 months, 59 (24.4%) in the intervention group vs. 60 (29.6%) in the control group, *p* = 0.22; 6–12 months, 14 (5.8%) vs. 12 (5.9%), *p* = 0.96; 12–24 months, 16 (6.6%) vs. 15 (7.4%), *p* = 0.75). Among the patients who were hospitalized at baseline, the number of patients hospitalized during the follow-up periods was also similar between the groups (*p* = 0.17, *p* = 0.95, *p* = 0.35, respectively). The median number of days spent in hospital was analyzed for patients hospitalized during the respective follow-up periods. At 0–6 months, the median number of days hospitalized was 16 (IQR 17) in the study group (*n* = 59) and 18.5 (IQR 16.75) in the control group (*n* = 60), at 6–12 months it was 17 (IQR 12.75) (*n* = 14) and 19 (IQR 29.75) (*n* = 12), and at 12–24 months it was 10 (IQR 12.25) (*n* = 16) and 21 (IQR 31) (*n* = 15).

### Dropout and deaths

There were no between-group differences in the number of patients who dropped out in any period (*p* = 0.79, *p* = 0.86, *p* = 0.51, respectively). There were four deaths in the intervention group and seven deaths in the control group (mortality rate: 1.7% vs. 3.4%, *p* = 0.23). Among these, the median time of death from baseline was 16 months (IQR 16) in the intervention group and 5 months (IQR 13) in the control group. We have no data on specific causes of death.

## Discussion

The study setting, a benchmark controlled trial, allowed the evaluation of BA treatment among ordinary patients in a routine clinical setting of secondary psychiatric care. The findings provide essential information for the planning and development of a better treatment protocol for depressed patients. Since only patients with psychotic or organic pathologies were excluded, our study group was heterogeneous with various comorbidities and therefore representative of the usual patient population in everyday practice. The depressive symptoms among the intervention group patients seem to alleviate compared to baseline during the 2-years follow-up. Our results suggest that BA is a useful tool for this patient group although strong conclusions can’t be drawn about the benefits compared to TAU. The intervention patients showed a greater improvement in functional ability than the control patients. This is an important finding, because functional recovery has a considerable effect on daily life and is particularly relevant from the patients’ point of view. The ODS intervention did not change the need for outpatient visits or hospitalizations compared with TAU. This finding is surprising, since one could assume that the positive change in functional ability would reflect in a reduced need for inpatient treatment in the intervention group. The dropout rates indicated that adherence to treatment was similar in both groups.

In a review of studies of the impact of homework in cognitive-behavioral therapy, Thase and Callan concluded that homework adherence increases the possibility of successful outcomes when treating depression [27]. This is one reason why BA was selected as an ODS treatment method instead of other evidence-based brief therapies. As BA aims to activate the patient (e.g., by homework and skills training) this may positively influence the overall functional ability of patients, resulting in functional recovery. The cost-effectiveness and outcomes of BA have been compared with those of more comprehensive cognitive-behavioral therapies in depressed adult patients and evidence indicates that BA is equally effective [28]. This suggests that brief interventions should be used at least in the public services, where the challenge is to provide effective treatment for a large number of patients despite limited resources. BA can be delivered successfully by mental health workers with no long-term training in psychological therapies [29] In the ODS, mental health workers with various backgrounds received short-term training in BA and delivered the intervention successfully. This indicates that this therapy could enhance the treatment of depression in the existing psychiatric health care system.

There are some limitations that should be considered when interpreting our results. BCTs are novel experimental designs which have yet to prove their methodological validity. Therefore, when considering the recommendations deriving from our paper it should be noted that this is a method that still needs to be validated against RCT (in this case BA vs treatment as usual groups). Data on intervention group patients was collected prospectively and data on control group patients was collected retrospectively. Data on intervention group patients was more comprehensive compared to the control patients. This is due to the inferior quality and the content of clinical information in the patient registers of the control group and it was not possible to complete the data afterwards. For example, it is possible that part of the information on somatic diagnoses of control patients is missing. We were unable to compare alleviation of depressive symptoms between the groups because of lack of information on BDI or MADRS scores for the control patients. Therefore, we could explore the effect of BA on the depression only in the intervention group. This is clearly a limitation when assessing our results in terms of the treatment of depressive symptoms. There was a slight between-group difference in baseline GAF scores. Thus, it was not possible to match the patients according to GAF levels at baseline. It could be argued that there is much inter-rater variation in GAF scores [30, 31] and that our results concerning GAF are therefore less reliable. We attempted to avoid this variation in scores using the calibration process described in section “Baseline assessment”. Intervention group patients with comorbid alcohol use problems also received MIs (a maximum of five appointments), which was mainly targeted at engaging patients with the treatment and increasing the success of BA. This practice was chosen according to the strong evidence of MI for substance use disorders [25]. As the use of MI may have had an additional positive impact on the treatment of depressive symptoms among the patients with alcohol use problems, the trial can be regarded to evaluate not only BA, but also the combination of BA and MI in this group. We did not screen for possible personality disorders at baseline. Although 13.6% of the cohort patients had other diagnosis from mood disorders as primary research diagnoses, they all had depressive symptoms with at least moderate severity and only few of these patients were clinically diagnosed in the personality disorders category. When further exploring the register based clinical diagnoses of personality disorders, approximately 10% of the total sample were diagnosed in this category. As it is known, personality disorders have a close effect on treatment response. Therefore, it should be noted that personality disorder as a secondary psychiatric diagnosis was more common in the control group relative to the intervention group (9 patients (3.7%) vs. 17 patients (8.4%). Also, the antipsychotic exposure was higher in the control patients, suggesting the control group may have more complex depressive illness than the intervention group. It was anticipated that patients with serious complicating factors, such as personality problems, would not benefit sufficiently from BA and could then be assessed more thoroughly and offered more comprehensive intervention.

## Conclusions

The systematic use of BA among secondary psychiatric care depressive patients provides encouraging results despite the patients had various comorbid non-psychotic disorders. Depressive symptoms alleviated and there was a trend towards better functional recovery among patients treated with BA compared to those treated with TAU in this study.

## Abbreviations

AUDIT: Alcohol Use Disorders Identification Test
BA: behavioral activation
BDI: Beck Depression Inventory
GAF: Global Assessment of Functioning scale
IQR: interquartile range
MADRS: Montgåmery-Åsberg Depression Rating Scale
MINI: Mini International Neuropsychiatric Interview
ODS: The Ostrobothnia Depression Study
SD: standard deviation
TAU: treatment as usual
